# Neural Pattern Classification Tracks Transfer-Appropriate Processing in Episodic Memory

**DOI:** 10.1523/ENEURO.0251-18.2018

**Published:** 2018-08-23

**Authors:** Inês Bramão, Mikael Johansson

**Affiliations:** 1 Department of Psychology, Lund University, Lund SE-221 00, Sweden

**Keywords:** EEG, episodic memory, multivariate pattern analysis, transfer-appropriate processing

## Abstract

The transfer-appropriate processing (TAP) account holds that episodic memory depends on the overlap between encoding and retrieval processing. In the current study, we employed multivariate pattern analysis (MVPA) of electroencephalography to examine the relevance of spontaneously engaged visual processing during encoding for later retrieval. Human participants encoded word-picture associations, where the picture could be a famous face, a landmark, or an object. At test, we manipulated the retrieval demands by asking participants to retrieve either visual or verbal information about the pictures. MVPA revealed classification between picture categories during early perceptual stages of encoding (∼170 ms). Importantly, these visual category-specific neural patterns were predictive of later episodic remembering, but the direction of the relationship was contingent on the particular retrieval demand of the memory task: a benefit for the visual and a cost for the verbal. A reinstatement of the category-specific neural patterns established during encoding was observed during retrieval, and again the relationship with behavior varied with retrieval demands. Reactivation of visual representations during retrieval was associated with better memory in the visual task, but with lower performance in the verbal task. Our findings support and extend the TAP account by demonstrating that processing of particular aspects during memory formation can also have detrimental effects on later episodic remembering when other aspects of the event are called-for and shed new light on encoding and retrieval interactions in episodic memory.

## Significance Statement

Episodic memory allows us to mentally travel in time to relive our personal past. The likelihood that a previous episode will be remembered is considered to depend on the extent to which processes engaged during encoding are also engaged at the time of retrieval. In the present study, we leveraged multivariate pattern analysis (MVPA) of high time-resolution oscillatory brain activity to assess participants’ category-specific visual processing during memory formation, and later cortical reinstatement at retrieval. The results demonstrate that category-specific visual processing may predict both benefits and costs in episodic memory, depending on the retrieval requirements. These novel findings elucidate the neurocognitive dynamics of encoding and retrieval, which mediate the access to our personal past.

## Introduction

Episodic memory allows us to revisit the past and to re-experience previous events from a first-person perspective (Tulving, 1983). Such re-experiencing is considered to be mediated by the reinstatement of the brain activity that was present during processing of the original event (Marr, 1971). The contents of memory depend on what type of information was at the focus of attention during encoding, which explains how different people may remember the same event differently (Conway, 2009) and how the relevance of a memory for guiding future behavior depends on whether the encoded information is applicable in times to come. The current study used a novel approach to investigate the relationship between processing engaged during encoding and later retrieval demands. We employed multivariate pattern analysis (MVPA) of oscillatory brain activity to examine the relevance of spontaneously engaged category-specific visual processing during encoding for later memory retrieval. By manipulating retrieval demands over two memory tasks, we here demonstrate that the particular attentional focus adopted during the encoding of an event can both facilitate and hinder performance on subsequent tests of memory for that event.

The transfer-appropriate processing (TAP) account holds that the likelihood of successful episodic memory depends on the extent to which the processing engaged by a retrieval cue overlaps with that engaged at encoding (Morris et al., 1977; Roediger et al., 1989). A successful retrieval cue is thought to trigger the reinstatement of the cortical patterns active at the time of encoding (Marr, 1971; Norman and O´Reilly, 2003). A growing body of research has consistently provided evidence for this notion by showing that successful retrieval co-varies with the replay of the encoding-related brain patterns at the time of retrieval (Nyberg et al., 2000; Wheeler et al., 2000; Polyn et al., 2005; Rugg et al., 2008; Manning et al., 2011; Staresina et al., 2012a; Gordon et al., 2014).

Furthermore, previous studies have shown that encoding-related brain activity (i.e., predictive of subsequent memory) is shaped both by the type of processing occurring at encoding and by the overlap between encoding and retrieval processes (Bauch and Otten, 2012; Fellner et al., 2013; Staudigl and Hanslmayr, 2013; Vogelsang et al., 2016; 2018; Long and Kahana, 2017). The typical procedure in these previous studies has been to investigate encoding-related brain activity when explicitly instructing participants to attend to particular attributes during the encoding and retrieval. In the current study, participants were not directed to process specific aspects of the stimuli, instead we used measures of brain activity to track their spontaneously adopted attentional focus during encoding and examine the extent to which processing during encoding matched later retrieval demands.

Participants learned paired-associates formed by a word and a picture from one of three different categories (famous faces, landmarks, and objects). Retrieval demand was manipulated in two memory tasks in which participants were cued by the word and instructed to recall either visual (orientation) or verbal (name) information about the associated picture. Previous work involving electroencephalography-based MVPA decoding of picture categories has shown that classification relies on early brain activity related to low-level visual processing involving category-specialized brain regions (Jafarpour et al., 2014; Kaneshiro et al., 2015; Kurth-Nelson et al., 2015). This allowed us to use classification accuracy as a proxy for spontaneously engaged visual processing during picture encoding. Thus, high classification accuracy in our MVPA approach indicates attention directed toward visual features of the pictures. To examine how category-specific visual processing during encoding transfers to and matches with later retrieval demands, the accuracy of the pattern classifiers was related to performance in the two memory tasks. Moreover, the classifiers established at encoding were used to decode the oscillatory brain activity during retrieval, when only the word cue was presented, which allowed us to examine cortical reinstatement and its functional significance depending on retrieval demand. In this novel paradigm, we expected TAP to be revealed in a positive relationship between classification accuracy and episodic remembering in the visual rather than in the verbal memory task, and further that the replay of encoding brain activity would occur and co-vary with performance in the visual memory task exclusively.

## Materials and Methods

### Participants

Thirty-six participants took part in the study in exchange for a movie ticket. Eighteen participants took part in the visual memory task (six males, average 23 years old, range 20–28) and the remaining 18 took part in the verbal memory task (six males, average 24 years old, range 21–40). An independent-sample *t* test confirmed that the two groups of participants were matched in age (*t*_(34)_ = 1.13, *p* > 0.25). All participants were right-handed, native Swedish speakers, and reported no history of neurologic diseases. The study was conducted in accordance with the Swedish Act concerning the Ethical Review of Research involving Humans (2003:460). Participants gave their written informed consent, and the study followed the local ethical guidelines at Lund University.

### Stimulus material

**Figure 1.**
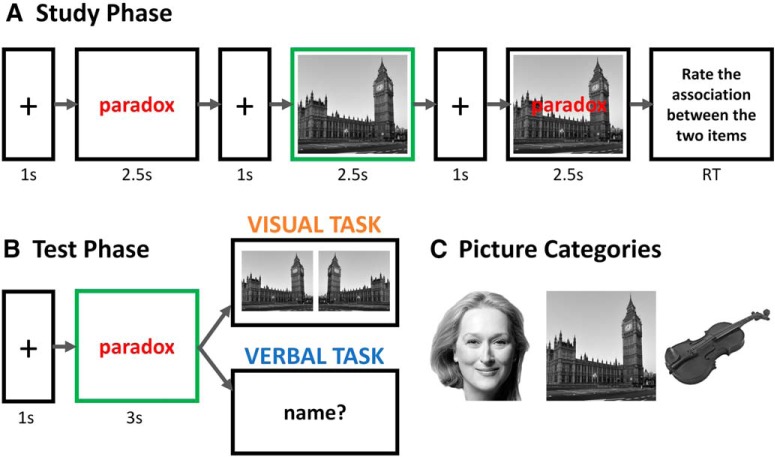
***A***, Trial structure during study. Notice that study phase was identical in the visual and the verbal memory tasks. The classifiers were trained and tested for decoding the picture category (face, landmark or object) based on the EEG TFRs (4–45 Hz) at different time bins when the picture was presented alone (outlined in green). ***B***, Trial structure during retrieval for the visual and the verbal memory tasks. Replay of the encoding category-specific neural patterns was examined during the presentation of the word cue (outlined in green). ***C***, Exemplars of the stimuli used in the paradigm.

A total of 192 words were selected from a comprehensive Swedish language corpus (Borin et al., 2012). Abstract words with low frequency were selected to limit visual imagery evoked by the word cue. The words were divided into three sets of 64 words each, ensuring that they were matched for length, frequency, and concreteness (*p* > 0.25). Additionally, 192 photographs of three different categories were used (64 faces, 64 landmarks and 64 objects; Fig. 1*C*). The photographs of faces consisted of well-known males (e.g., Tom Hanks) and females (e.g., Meryl Streep). Landmarks consisted of well-known locations, including natural landscapes (e.g., Niagara Falls) and man-made structures (e.g., Big Ben). Objects consisted of everyday man-made objects (e.g., violin, axe). All photographs were converted to a black-and-white format with 600 × 600 pixels and with a resolution of 72 pixels/inch. Each word was assigned to one picture from each category, with no obvious pre-experimental association between word and pictures. Three stimulus sets were created that counterbalanced the word-picture selection across participants.

### Experimental design and procedures

The memory task comprised eight blocks, each including a study phase, a distractor task and a test phase. In each block, participants were asked to memorize 24 paired-associates composed of a cue word and a target picture that could be either a face, a landmark or an object. In the visual memory task, participants were instructed to retrieve the semantic category of the picture, followed by a forced choice about two versions (original vs mirrored) of the picture that was studied with the word. Participants indicated their response by pressing a response button. In the verbal memory task, participants were instructed to verbally retrieve the semantic category and the name of the picture studied with each word. Except for the retrieval demand differences in the test phase, the encoding phase was identical in the two tasks (Fig. 1*A*). Each encoding trial started with a 1-s fixation cross followed by the presentation of the word cue in red for 2.5 s. Next, the target image was presented for 2.5 s, preceded and followed by a 1-s fixation period. Immediately thereafter, the target picture appeared with the red word cue presented on top and remained in the screen for another 2.5 s. Each trial ended with participants rating how easy it was to associate the word cue with the target picture (1 = very difficult, 2 = OK, 3 = very easy). The trials were randomly presented with the constraint that consecutive trials were from different categories.

To eliminate active rehearsal of the last associate pair, an arithmetic task separated the study from the test phase. At test, each trial started with a fixation period of 1 s followed by the display of the word cue for 3 s (Fig. 1*B*). In the visual memory task, participants selected the category of the target picture associated with the word cue (1 = face, 2 = landmark, 3 = object, or 4 = don’t know). If they were correct, participants were asked to make a forced-choice decision about which version of the picture was presented during encoding (1 = left and 2 = right). The assignment of the buttons to category and target picture selection was counterbalanced across participants. In the verbal memory task, participants were asked to verbally retrieve the semantic category and the name of the exemplar in the target picture (e.g., Big Ben) associated with the word cue. To avoid muscle artifacts in the electroencephalogram (EEG) recordings, participants were instructed to withhold their response during the presentation of the word cue. If they did not remember the exact name, participants were encouraged to tell everything they remembered about the picture (e.g., “the clock in London”). In both tasks, each trial was separated by an interstimulus interval of 0.5 s.

To ensure that participants were familiar with all the stimulus material, previous to the EEG recordings, participants were familiarized with each picture and respective name. Each image was displayed on screen for 2.5 s, followed by its name for 1 s and participants were asked to rate how familiar they were with each of the identities represented by the picture using a five-point scale (1 = not familiar and 5 = very familiar).

### EEG recording

The EEG was recorded continuously using a Neuroscan (Compumedics) Grael amplifier (2048-Hz sampling rate; left mastoid reference) from 31 Ag/AgCl scalp electrodes mounted in an elastic cap and positioned according to the extended 10–20 system. The montage included five midline electrode sites (Fz, FCz, Cz, Pz, Iz) and 13 sites over each hemisphere (FP1/FP2, F7/F8, F3/F4, FC5/FC6, FC1/FC2, T7/T8, C3/C4, CP5/CP6, CP1/CP2, P7/P8, P3/P4, PO9/PO10, and O1/O_2_). Additional electrodes were used as ground (AFz), reference sites (mastoids), and for recording the electrooculogram (EOG). EOG electrodes were placed below the left eye and at the left and right outer canthi.

### EEG preprocessing and data preparation

The EEG data were preprocessed using FieldTrip (Oostenveld et al., 2011). Offline, the data were downsampled to 512 Hz, and divided into epochs of 4 s, from -1.5 to 2.5 s relative to both the onset of the word cue and target image in the study phase and relative to the word cue in the test phase. The data were transformed to a linked-mastoid reference, and a baseline correction was applied (subtraction by the average amplitude of the epoch; as in Jafarpour et al., 2013, 2014). Bipolar EOG was computed using the FP1 and the electrodes placed vertically and horizontally around the eyes. EEG epochs were physically inspected and those containing muscle or other artifacts, not related to blinks and horizontal eye movements, were manually removed. Independent components analysis was conducted and components representing blink and other oculomotor artifacts clearly distinct from EEG were removed and bad channels (if any) were interpolated. The data were thereafter again visually inspected and trials with residual artifacts were manually excluded.

The signals from individual trials were transformed into time-frequency representations (TFRs). Brain oscillatory activity from the low θ range to the high γ frequency represents core mechanisms of episodic memory (Hanslmayr et al., 2016) and have been previously used for training pattern classifiers to distinguish early visual encoding brain representations correspondent to faces and visual scenes (Jafarpour et al., 2014). TFRs were obtained for frequencies ranging from 4 to 45 Hz, with a frequency step of 1 Hz, a time step of 0.01 s, and a wavelet width of five cycles, using the complex Morlet wavelet transform as implemented in FieldTrip.

### Multivariate pattern classification and statistical analysis

Classification analysis was used to build pattern classifiers that could distinguish the encoding brain activity related to the three picture categories (faces, landmarks, and objects). MVPA was performed using support vector machine (SVM), with a linear kernel, and a one-against-all strategy, as implemented in the MATLAB bioinformatics toolbox. The pattern classifiers were trained on the TFR elicited when the target picture was shown alone during the encoding phase (Fig. 1*A*). Twenty different classifiers were trained separately for each participant. The 20 classifiers were trained using 20 different time bins that covered a time period from -45 to 920 ms after picture onset. The 20 time bins, each with a duration of 39 ms (spanning over five time points), were centered at -25, 23, 72, 121, 170, 219, 268, 316, 365, 414, 463, 512, 561, 609, 658, 707, 756, 805, 854, and 902 relative to stimulus onset, to cover the whole epoch. Each of the classifiers used the TFR in the 31 EEG channels and in five time points within each time bin. Thus, for each classifier, there were 6510 possible features (31 channels × 42 frequencies × five time points). No additional baseline correction was performed on the TFR and instead the power at each timepoint, frequency, and channel was normalized across trials (as in Jafarpour et al., 2013, 2014). Each classification used a ten-fold cross validation, that is, the data were randomized and partitioned into ten rough equal-sized subsets, over which ten training-test iterations were performed. Each partition was used as the test set exactly once, with the remaining nine partitions used for training the classifier in that fold. In the visual memory task, classification was performed using the TFR signal of an average of 62 trials corresponding to faces (range 60–64), 62 corresponding to landmarks (range 59–64), and 62 corresponding to objects (range 58–64). In the verbal memory task, analysis was performed on an average of 63 trials corresponding to faces (range 60–64), 62 corresponding to landmarks (range 57–64), and 62 corresponding to objects (range 57–64). Before the classification, in each cross-validation iteration, a feature-selection step was performed by calculating a univariate statistical test across the training subset (excluding the test subset) on spectral power at each frequency, time point, and channel that constituted the features of the classifier. The features that were found to be significantly different between categories using a one-way ANOVA (*p* < 0.05) were selected for classifier training and z-transformed. In each cross-validation iteration, the model was used to predict the category of the left-out trials (i.e., test subset). Thus, the classification accuracy here reported represents the performance of the classifier averaged over categories (faces, landmarks, and scenes), cross-validation iterations, and participants. Classification performance for target picture was contrasted against classification performance for word cue at study. During word cue presentation, the pattern classifiers do not carry information about the specific stimulus category, and the word cue therefore offers a perfect baseline control condition. The data for this baseline classification analysis was preprocessed and treated in the same way as described above for the target picture classification. To account for multi-comparisons problem the significance level of each test was Bonferroni corrected (corrected *p* value: 0.05/20 classifiers = 0.0025).

The pattern classifiers built during encoding were used to predict retrieval without any tuning to optimize cross-validation performance. The testing was performed at 20 separate time bins centered at -25, 23, 72, 121, 170, 219, 268, 316, 365, 414, 463, 512, 561, 609, 658, 707, 756, 805, 854, and 902 relative to word cue onset (the same time bins as for the encoding phase). The classification accuracy was calculated in relation to the category of the target picture (i.e., the to-be-retrieved picture). We looked at replay in trials for which participants successfully retrieved the target. To generate the statistical significance of the replay, a permutation test procedure, with 500 iterations, was used to generate the null distribution for the significance thresholds. In each iteration, the labels for the stimulus category were shuffled. Thus, each iteration yielded a distribution that contained no true information about the category of the picture but preserved overall smoothness and other statistical properties. At each iteration a one-sample *t* test comparing classification performance against chance (33.3%) was conducted. The distribution of the *t* tests formed the nonparametric empirical null distribution and the 95th percentile of this distribution is reported as the significance threshold. All study (target picture onset) × retrieval (word cue onset) maps of classification performance were smoothed by a 2-D Gaussian kernel spatial filter with a σ of 1 for display and for calculating the *t* test of the non-parametric distribution.

To investigate the contribution of each channel for classification accuracy, we re-run the classification training at study and the category prediction at test using one channel and its neighbors at a time and storing the classification performance at the center channel. to compare the classification topography between the two tasks, the normalized mean classification at each channel was compared between the visual and the verbal task. Bonferroni correction was performed for correction of multiple comparisons (corrected *p* value: 0.05/31 channels = 0.0016).

### Relationship between pattern classifier accuracy and retrieval demands

The relationship between classifier accuracy during encoding and episodic remembering was tested using the Robust Correlation Toolbox in MATLAB (http://sourceforge.net/projects/robustcorrtool/). First, we correlated the highest classifier accuracy during encoding with memory performance in the verbal and visual memory tasks. We calculated the Person correlation and the robust 95% confidence intervals (CIs) which are computed by bootstrapping the data after removing outliers to prevent them from exerting disproportionate leverage (Pernet et al., 2013). Correlations were considered significant if the 95% CI did not include zero.

We also correlated the accuracy obtained in each classifier during encoding with memory performance to evaluate if the correlation was also present in the neighboring time bins. To correct for multiple comparisons, a permutation test procedure (50,000 iterations) was used to generate a null distribution for the significance thresholds. In each iteration of the test, we randomly scrambled the order of the subjects (thereby eliminating any inherent correlation) and the correlations were recomputed. Next, the resulting Pearson *r* values corresponding to the 95th and 5th percentiles were calculated and used for the significance threshold. For display, the resulting Pearson *r* values were smoothed by a 2-D Gaussian kernel spatial filter with a σ of 1.

Classification accuracy during retrieval was also correlated with memory performance. For this, we used Person *r* correlations with a two-tailed level of significance.

### Encoding-related time-frequency analysis

We investigated the subsequent memory effect (SME) in the time window where a significant relationship between encoding pattern classification and episodic remembering was observed. The data from encoding were preprocessed in the same way as described for the pattern classification. The TFR was averaged separately for successful and non-successful memory retrieval and the power estimates for each time point were logtransformed and baseline corrected by the average power in a -0.5- to 0-s time window relative to onset of the target picture.

The statistical significance of these effects was conducted using a nonparametric cluster- based permutation test implemented in FieldTrip (Maris and Oostenveld, 2007). In a first step, a dependent-samples *t* test is performed to compare the conditions (successful and non-successful target retrieval) and identify statistically significant data samples (α = 0.05). All adjacent data samples (either spatial or temporal neighbors) are then grouped into clusters and the *t* values within each cluster are summed and used to generate a cluster-level *t* value. The type-1 error rate for the complete data matrix is controlled by evaluating the cluster-level test statistic under the randomization null distribution of the maximum cluster-level test statistic. This was obtained by randomizing the data between conditions for each participant. By creating a reference distribution from 6000 random draws, the *p* value was estimated according to the proportion of the randomization null distribution exceeding the observed maximum cluster-level test statistic (the so-called Monte Carlo *p* value). In this way, significant clusters extending over time, frequency, and electrodes can be identified.

Furthermore, to investigate whether the underlying neural mechanisms supporting these effects in the visual and the verbal memory task were different, a topographical analysis of the observed effects was conducted. The TFR of the effects (successful vs unsuccessful target retrieval) on a set of representative sites (F7/F3/Fz/F4/F8, T7/C3/Cz/C4/T8, and P7/P3/Pz/P4/P8) was subjected to a repeated-measures ANOVA, including the factors memory task (visual vs verbal), region (frontal vs central vs posterior), and hemisphere (left peripheral vs left central vs midline vs right central vs right peripheral). The data were vector scaled (McCarthy and Wood, 1985), and Greenhouse–Geisser corrections were applied when appropriate (Greenhouse and Geisser, 1959).

## Results

### Behavioral results

For each task and participant, we calculated the percentage of exemplar and category hits. In the visual memory task, a given trial was considered a category hit if participants correctly identified the picture category and a target hit if they additionally identified the original picture. Overall, participants were successful identifying the category of the target 69 ± 13% (mean ± SD of category hits). Of these trials, 74 ± 6% (mean ± SD) corresponded to exemplar hits. A one-sample *t* test confirmed that participants were able to identify the original target picture significantly above the 50% chance level (*t*_(17)_ = 16.7; *p* < 0.001).

For the verbal memory task, a trial was considered an exemplar hit if participants correctly named the exemplar in the target picture or alternatively, provided rich details of it (e.g., “the clock that is in London” or “the actress in the Mamma Mia movie”). In general, participants successfully recalled the name of the target picture 53 ± 16% (mean ± SD) of the times. A category hit was a trial for which participants provided evidence of knowing the category of the associated picture. Thus, trials in which participants answer with the category name but did not retrieve any detail of the specific identify represented by the picture were accounted as a category hit (e.g., a “place in a city” or a “face of female”). Overall, participants provided evidence of knowing the category of the target 62 ± 18% (mean ± SD).

Pairwise comparisons showed that the percentage of exemplar hits was higher in the visual compared with the verbal memory task (*t*_(34)_ = 5.35; *p* < 0.001), revealing that it is easier to retrieve the picture than the name of the identity represented by the picture. However, there was no differences in the percentage of category hits between the two memory tasks (*t*_(34)_ = 1.33; *p* = 0.194). This indicates similar behavioral performance between the tasks when the retrieval requirement is the semantic category.

We also investigated whether participants were better at retrieving information from one specific category (Table 1). A repeated-measures ANOVA including the factors task (visual vs verbal) and category (faces vs landmarks vs objects) was run separately for exemplar and category hits. The analysis of the exemplar hits revealed significant main effects of task (*F*_(1,34)_ = 31.03; *p* < 0.001; η^2^ = 0.48) and category (*F*_(2,68)_ = 8.24; *p* = 0.001; η;^2^ = 0.20) and also a significant two-way interaction between the two factors (*F*_(2,68)_ = 19.43; *p* < 0.001; η;^2^ = 0.36).

**Table 1.** Mean ± Standard Deviations for Exemplar and Category Hits, shown for each memory task and stimulus category.

		Total	Faces	Landmarks	Objects
Exemplar hits	Visual	74 ± 6%	66 ± 9%	79 ± 8%	80 ± 7%
	Verbal	53 ± 16%	55 ± 16%	46 ± 17%	55 ± 20%
Category hits	Visual	69 ± 13%	76 ± 13%	70 ± 15%	62 ± 16%
	Verbal	62 ± 18%	69 ± 17%	60 ± 19%	58 ± 19%

Pairwise comparisons showed that for the visual memory task participants were worse at selecting the correct associated picture when the picture was a face compared with both landmarks tasks (*t*_(17)_ = -5.1; *p* < 0.001) and objects tasks (*t*_(17)_ = -7.7; *p* < 0.001). For the verbal memory task, participants were worse at recalling the names of the landmarks compared with both the faces (*t*_(17)_ = -3.2; *p* = 0.005) and objects (*t*_(17)_ = -3.1; *p* = 0.006). In terms of category hits, the analysis also revealed a main effect of category (*F*_(2,68)_ = 38.1; *p* < 0.001; η;_*p*_ = 0.53). Participants were better at retrieving the category if the image was a face compared with both landmarks (*t*_(35)_ = 5.3; *p* < 0.001) and objects (*t*_(35)_ = 9.0; *p* < 0.001), and they were better at retrieving landmarks compared with objects (*t*_(35)_ = 3.2; *p* = 0.002). However, no significant effect of task (*F*_(1,34)_ = 1.8; *p* = 0.18; η;_*p*_ = 0.05) nor interaction between task and category (*F*_(2,68)_ = 1.7; *p* = 0.18; η;_*p*_ = 0.05) was observed. In sum, although we identified differences in memory performance as a function of category between the two tasks when the exemplar hits were analyzed, these differences disappeared when the nature of the response between the two tasks is matched, that is, when category hits are analyzed.

A final analysis revealed that participants’ rated associative strength was comparable between the two tasks, in total (visual = 1.7 ± 0.3; verbal = 1.6 ± 0.3; *t*_(34)_ = 0.94; *p* = 0.352) and also for each stimulus category (faces: *t*_(34)_ = 1.59; *p* = 0.120; landmarks: *t*_(34)_ = 0.76; *p* = 0.455; and objects: *t*_(34)_ = 0.37; *p* = 0.714).

### EEG-based decoding tracks early visual processing during encoding

**Figure 2.**
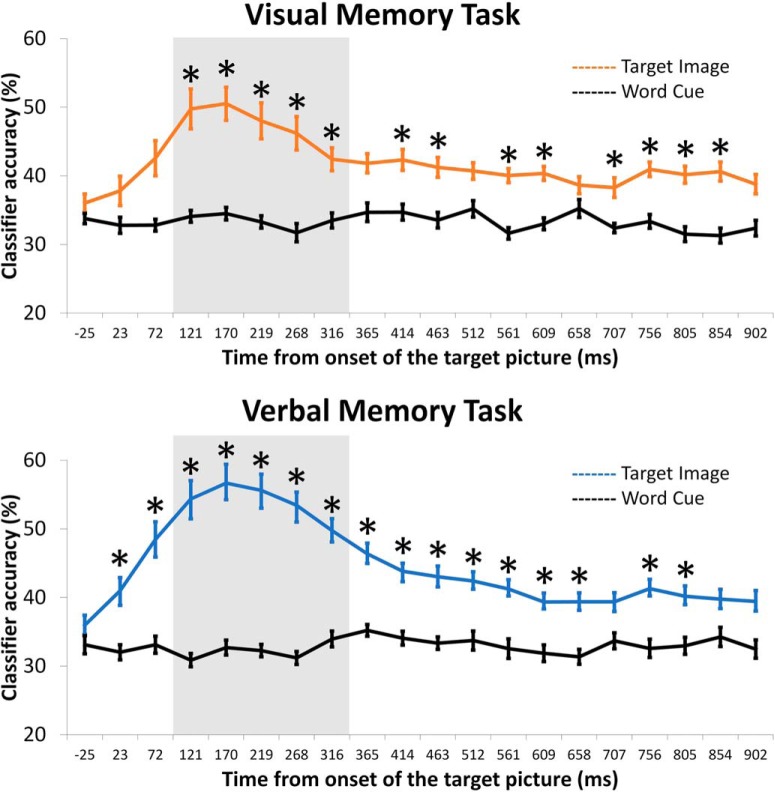
Averaged accuracy of the 20 cross-validated pattern classifiers trained to discriminate between faces, landmarks, and objects during the encoding phase of the visual and of the verbal memory task. The colored line represents the accuracy of the classifiers trained during the presentation of the target picture. The black line shows the baseline accuracy of the classifiers trained during word cue presentation. Highlighted (*) are the times bins for which classification performance survived multiple comparison correction. In gray is highlighted the time bins for which classification accuracy was highest.

To quantify the visual processing during encoding, we trained pattern classifiers to decode the brain activity related to picture encoding. Twenty pattern classifiers were trained at different time bins from -45 to 920 ms after picture onset, and their performance was compared with a baseline classification after word cue onset. Compared with baseline, classifiers trained on picture-related activity showed significant classification accuracy in both memory tasks (all *p*s < 0.05). The classifiers that survived correction for multiple comparisons are highlighted (*) in Figure 2 separately for the visual and verbal memory tasks. An early peak between 120 and 320 ms after picture onset emerged from the classification in both tasks. The classifier with highest accuracy was centered at ∼170 ms after picture onset for both the visual (mean ± SD = 51 ± 11%) and the verbal (mean ± SD = 57 ± 12%) memory tasks. A direct comparison of the classification accuracy between the two tasks indicated comparable classification accuracy in the two memory tasks.

**Figure 3.**
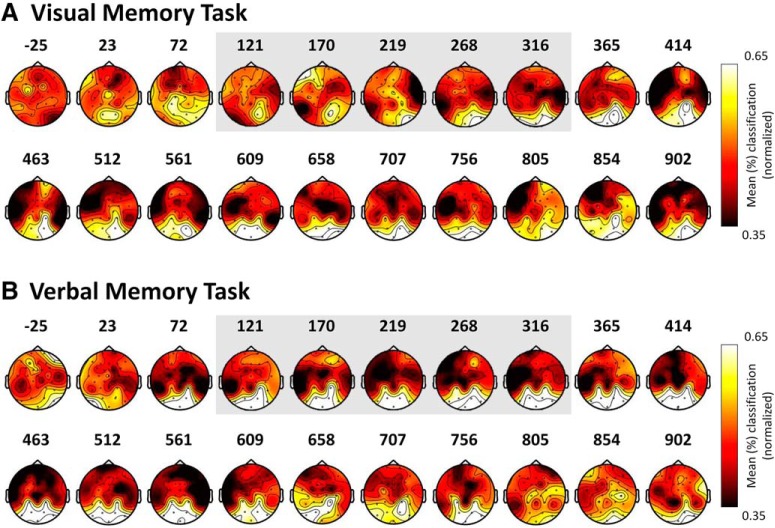
Contribution of each channel to the performance accuracy of the 20 pattern classifiers during the encoding phase of the visual and the verbal memory task. The gray frame highlights the time bins for which classification accuracy was highest.

MVPA classification of brain activity in this early time window has previously been linked to visual processing necessary to distinguish different categories of stimuli (Jafarpour et al., 2014; Kaneshiro et al., 2015; Kurth-Nelson et al., 2015). to substantiate this and provide further evidence that classification accuracy in the present study can be used as proxy for visual processing occurring during encoding, we investigated the channel contribution to classification performance of the 20 pattern classifiers trained during picture encoding. Confirming that classification accuracy is based on visual processing, the analysis showed that posterior channels contribute the most to classification accuracy in both tasks (Fig. 3). Interestingly, even in later time windows the posterior channels show the highest contribution to classification performance, indicating that classification performance is based on continued visual processing throughout all the epoch. The topographies were statistically indistinguishable in the two memory tests.

In summary, the pattern of the MVPA classification is, in terms of both time and topography, similar in the visual and verbal memory tasks, indicating that classification accuracy reflects visual processing operations in both tasks.

### Early visual processing during encoding interacts with later retrieval demands

**Figure 4.**
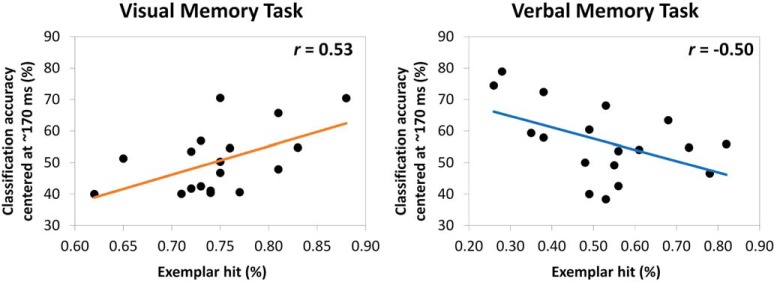
Relationship between the accuracy of the pattern classifier trained at 170 ms after picture onset during study and the proportion of exemplar hits in the visual and in the verbal memory task.

To test the prediction that visual processing at study would only be beneficial when the memory task requires the retrieval of visual information, we investigated the relationship between classification accuracy at study and later episodic memory performance. We selected the pattern classifier for which performance accuracy was highest (∼170 ms) and correlated its accuracy with successful memory performance (exemplar hits %) for both tasks. As expected, we observed a significant positive correlation for the visual memory task (*r* = 0.534 [0.07, 0,81]; *p* = 0.022; Fig. 4). In accordance with our predictions, participants who spontaneously directed attention to perceptual features of the stimuli, as indicated by pattern classification accuracy, were more likely to correctly identify the original target picture from encoding. This finding offers novel support for the TAP account in a paradigm that assessed the modulatory role of attention during encoding without explicitly directing participants to particular stimulus attributes. Interestingly, we also observed a negative association between visual processing at study and memory performance in the verbal memory task (*r* = -0.503 [-0.78, -0,11]; *p* = 0.038; Fig. 4), indicating that individuals who adopted a visual attentional focus during study were less likely to retrieve the correct name of the exemplar target picture. This negative correlation suggests that the allocation of resources to stimulus aspects that are not relevant for later retrieval demands (here, visual focus in a verbal memory task) is not only non-beneficial, as predicted, but can even be detrimental when other aspects are goal relevant (i.e., verbal, lexical information).

### Temporal profile of the association between processing during study and later retrieval demands

We next explored the temporal dynamics of the association between visual processing tracked by pattern classification at study and retrieval demands in the ensuing visual and verbal memory tasks. We predicted that the association between the visual processing tapped by the EEG-based decoding and memory retrieval should be seen not only for the classifier with the highest accuracy (∼170 ms) but also for its temporal neighbors. To do so, we correlated memory performance in terms of both successful (i.e., exemplar hit) and unsuccessful target retrieval (i.e., when participants completely failed to retrieve any information about the target picture; thus, trials for which participants only correctly retrieved the semantic category are excluded from this analysis) with the accuracy of all the 20 pattern classifiers (for details, see Materials and Methods).

**Figure 5.**
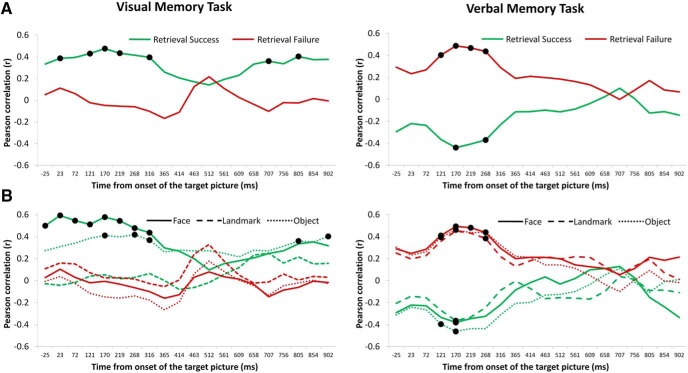
Pearson *r* values showing the temporal profile of the association between visual processing (tracked by EEG-based decoding) and memory performance in the visual and verbal memory task. Highlighted (•) are time bins for which the relationship with behavioral performance survived correction for multiple comparisons (for details, see Materials and Methods). ***A***, The green line shows the association for retrieval success (i.e., exemplar hits) and the red line shows the association for retrieval failure (i.e., when participants fail to retrieve any information about the target picture). ***B***, Association for retrieval success (green lines) separated for faces (solid green line), landmarks (dashed green line) and objects (dotted green line), and for retrieval failure (red lines) separated for faces (solid red line), landmarks (dashed red line), and objects (dotted red line).

Figure 5*A* shows the temporal profile of these associations in the two memory tasks. As predicted, the associations are not sporadic but extend to neighboring pattern classifiers. Interestingly, the accuracy of two pattern classifiers trained at later time bins (∼707 and ∼805 ms) also show a significant association with memory performance in the visual memory task. Figure 3 shows that these later pattern classifiers are also most likely based on visual processing, indicating that sustained visual processing is associated with a benefit in retrieving the originally encoded target picture in the visual memory task.

Moreover, for the verbal memory task we observed a positive relationship between the accuracy of early pattern classifiers and unsuccessful memory performance, indicating that participants with high levels of visual processing, tracked by classification accuracy, were not only less likely to correctly retrieve the name of the image but also more likely to fail at retrieval (Fig. 5*A*). This is a novel and interesting finding, corroborating the idea that focusing on task-irrelevant aspects of the stimuli during encoding may have a negative impact when later tests of memory requires retrieval of other aspects of the same event.

Next, the same analysis was conducted separately for each picture category. to evaluate if the profile of the observed association between classifier accuracy and memory performance is general or driven by one specific semantic category, we correlated classifier accuracy with memory performance for each of the individual categories. Figure 5*B* shows the temporal profile of this association. In general, the pattern of results remains when overall classifier accuracy is correlated with memory performance for each of the three categories of stimuli, indicating that the reported associations due not depend on a particular semantic category. However, no significant association for landmarks was observed for the visual memory task. One possible explanation for this result is that participants used some kind of verbal coding to memorize the landscape pictures (e.g., “the tower is in the right side of the image”), which was more difficult to adopt for faces and objects.

### Task-relevant memory encoding differs as a function of retrieval demands

**Figure 6.**
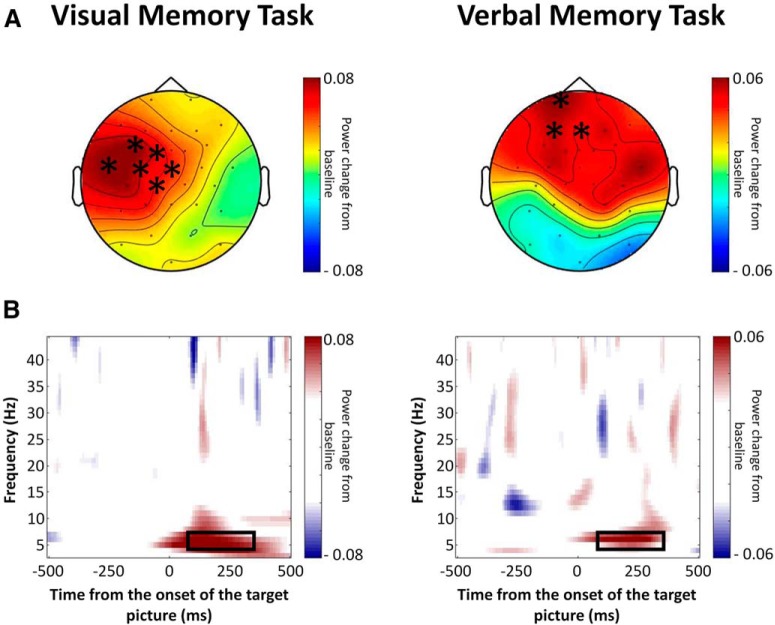
***A***, Topography of the SME (5–7 Hz) observed between 120 and 320 ms for the visual and the verbal memory task. Electrodes that reached significance in the cluster-based permutation test are highlighted (*). ***B***, TFRs from a representative channel for the visual (FC5) and verbal (F3) memory task.

The analyses above demonstrate that early visual processing during encoding is predictive of later memory performance depending on the particular retrieval demands of the test. A subsequent memory analysis was used to further investigate whether processing captured by the pattern classifiers is relevant for successful encoding. Additionally, because previous studies have shown that focusing on different aspects of the stimulus at encoding affects the resulting memory representation (Fellner et al., 2013), we predicted that the memory representation necessary to perform the upcoming retrieval task will differ as a function of the retrieval demands. An SME analysis was used to test the prediction that early memory formation (120–320 ms), when pattern classifier showed significant relationship with retrieval demands, would be different across the two memory tasks. A cluster permutation test was used to investigate the SME in the time period between 120 and 320 ms on the range of frequencies (4–45 Hz) used for pattern classifier training. This analysis identified no significant effects. However, when the analysis was constrained to the θ frequency range (5–7 Hz) we observed significant SMEs in both tasks (visual memory task: *p* = 0.04; verbal memory task: *p* = 0.05; Fig. 6). SMEs characterized by θ power increases are well documented in previous literature (Hanslmayr and Staudigl, 2014; Hanslmayr et al., 2016). Additional analyses were run in the α (8–12 Hz), β (13–30 Hz), and γ (30–45 Hz) bands, and no significant additional effects were observed (all *p*s > 0.19).

Crucially, a topographical analysis revealed a significant two-way interaction between the factors memory task and hemisphere (*F*_(4,136)_ = 2.78; *p* = 0.05; η;^2^ = 0.075), showing that the SME is supported by different neural generators in the two memory tasks. While the SME for visual memory task was left lateralized, the SME for the verbal memory task showed a more bilateral distribution (Fig. 6), confirming that the task-relevant memory representation formed already at this very early encoding stage varies as a function of the retrieval demands.

### Replay of encoding brain activity at retrieval differs as a function of retrieval demands

Next, we investigated whether the early category-specific neural patterns from encoding were replayed at any time during retrieval, as predicted from cortical reinstatement theory. Considering the observed association between classifier performance and retrieval demands, we expected the functional significance of the replay to be different in the two memory tasks. In the visual memory task, we predicted replay to be beneficial. Conversely, in the verbal memory task we predicted replay to be unrelated to performance or even detrimental given the observed negative association between pattern classification at study and retrieval performance in this task. The replay was tested during the word cue presentation at retrieval and for exemplar hit trials, that is, when participants correctly selected the original target picture in the visual memory task or remembered the name of the exemplar depicted target picture in the verbal memory task.

**Figure 7.**
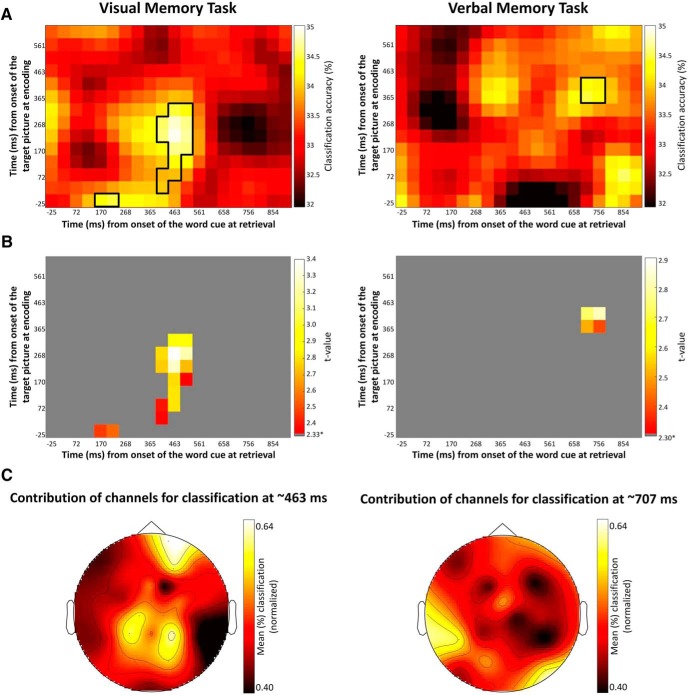
For the visual memory task, the classifiers trained between 120–320 ms after picture onset during study and tested around 450 ms after word cue onset at retrieval decode the stimuli category previously associated with the cue. For the verbal memory task, the classifiers trained between 350–450 ms after picture onset during study and tested around 700 ms after word cue onset at retrieval decode the stimuli category previously associated with the cue. ***A***, Accuracy of the decoding at retrieval for both tasks. The black outlines show p = 0.05 significance thresholds generated by a permutation test. ***B***, Results of comparing the accuracy of the replay against chance (33%). * Denotes critical t-value. ***C***, Contribution of each channel for the accuracy of the significant replay of the early visual pattern classifiers at retrieval for the visual and verbal memory task.

**Figure 8.**
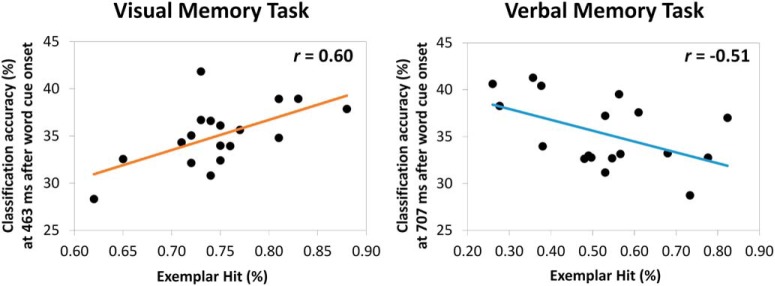
Relationship between the replay of the early visual pattern classifier at retrieval and the proportion of exemplar hits in the visual and in the verbal memory task.

The analysis revealed significant classification of the category of the images associated with the word cue elicited by the onset of the word at retrieval. For the visual memory task, we observed that the early category-specific activity during encoding (∼120–320) was replayed at 463–512 ms after word cue onset during memory retrieval (Fig. 7*A*,*B*). Consistent with our prediction, this neural replay was associated with an increase in memory performance (*r* = 0.60 [0.18, 0.87]; *p* = 0.009; Fig. 8). Additionally, the early pattern classifier trained at encoding at around -25 ms was replayed at 170–220 ms after word cue onset during retrieval. Note that the re-emergence of this classifier, trained in a pre-stimulus interval, is most likely due to poststimulus effects being temporally smeared into the prestimulus interval due to wavelet filtering. However, no significant functional relationship (*r* = 0.06 [-0.32, 0.50]) between this re-emergency effect and memory performance was observed which clouds interpretation of the effect.

We also observed significant replay in the verbal memory task. The visual classifiers identified during encoding (∼350–420 ms) were replayed later during retrieval (∼707 ms; Fig. 7*A*,*B*). Following our prediction, the re-emergence of these visual pattern classifiers at retrieval in the verbal memory task was negatively associated with memory performance (*r* = -0,51 [-0.80, -0.07]; *p* = 0.033; Fig. 8).

To further understand the nature of the replay of the encoding brain patterns at retrieval, a final analysis investigated the contribution of each channel to classification (using the replaying pattern classifiers that correlated with memory performance). The classification procedure was repeated for one channel and its neighbors at a time. Classification performance was recalculated and allocated to the central channel. Interestingly, the channels that showed the highest contribution to classification were not the posterior ones, and they were clearly different for the visual and the verbal memory task (Fig. 7*C*). Recent research has also provided evidence that the reinstated information at retrieval may be a transformed representation of the encoded information rather than a week replica of the encoded representation (Xiao et al., 2017). The results observed in the present study align well with these recent findings.

## Discussion

A prominent idea in the memory literature is that episodic remembering depends on the extent to which cognitive operations engaged during encoding match those engaged during retrieval (Morris et al., 1977; Roediger et al., 1989). The present study employed a novel experimental approach to investigate this principle of TAP, by capitalizing on MVPA of brain activity to track spontaneously engaged processing during encoding and to assess transfer depending on later retrieval demands. Our approach thus allowed participants to freely allocate and adjust their processing resources and attentional focus to whatever attribute they considered relevant. Category-specific neural patterns observed at encoding and replayed at retrieval were indeed predictive of later episodic remembering. These findings provide novel support for the TAP account and shed new light on the dynamics of encoding and retrieval.

In two memory tasks, with different retrieval demands, we used MVPA to decode, from the oscillatory brain data, category-specific representations of faces, landmarks, and objects. Our EEG-based decoding captured spontaneously engaged visual processing necessary to distinguish the different stimulus categories, presumably driven by category-specific brain regions along the inferior temporal cortex. We trained consecutive classifiers during picture presentation at encoding, which allowed us to track the development of these category-specific brain oscillatory representations over time. The early onset (∼170 ms) and the marked posterior topography (Figs. 2, 3) of these representations indicate that they reflect low-level visual processing necessary for the selection of the object models corresponding to faces, landmarks, and objects (Schendan and Kutas, 2007). Interestingly, these early visual category-specific patterns were not only associated with the predicted benefit when retrieving visual information about an event, but with a cost, when instead verbal information, the picture name, was demanded at retrieval (Figs. 4, 5). Specifically, early classification of visual processing predicted successful memory in the visual task and memory failure in the verbal task. These results indicate that participants who spontaneously focused their attention on the visual attributes of the pictures during encoding, were more likely to succeed in the visual memory task but, conversely, fail in the verbal task.

In line with previous research (Sederberg et al., 2003; Staudigl and Hanslmayr, 2013; Long et al., 2014), SMEs were observed in the θ band. Our results add weight to previous claims underscoring the important role of θ oscillations in the binding of contextual information supporting episodic memories, and the role of θ power in encoding-retrieval overlap (for review, see Hanslmayr and Staudigl, 2014). Interestingly, while the SME in the visual memory task was left lateralized, the SME observed in the verbal memory task was more widespread (Fig. 6). The different topographies indicate that encoding was supported by nonoverlapping neural generators, providing evidence that different memory representations formed at encoding are applicable in the two upcoming memory tasks. This finding underscores the modulatory role of attention during encoding and is consistent with predictions from the TAP account underscoring the processing overlap between encoding and retrieval. Notably, the encoding-related category-specific representations were evident already during very early encoding, between 120 and 300 ms after picture onset, when pattern classification performance was highest. Speculatively, it is conceivable that participants may spontaneously adjust their encoding to fit later retrieval demands. An interesting objective for future work is to examine how processing during encoding is modified over time, across multiple study-test blocks.

To examine cortical reinstatement during episodic remembering, the classifiers established during picture encoding were used to decode the oscillatory brain activity during retrieval, when only the word cue was presented. Our results demonstrate that visual category-specific processing was replayed during memory retrieval, and further that the timing and consequence for performance differed as a function of retrieval demands. In the visual memory task, the replay occurred relatively early (∼463 ms) after word cue onset and was predictive of successful retrieval, whereas the replay in the verbal memory task occurred later (∼707 ms) and was conversely associated with lowered memory performance (Figs. 7, 8). These replay results thus mirror those observed for the category-specific processing during encoding.

Long-term remembering is dependent on the processing occurring during both encoding and retrieval, and the greater the overlap between the cognitive operations that took place during encoding and retrieval the greater the likelihood of successful retrieval (Tulving and Thomson, 1973; Morris et al., 1977). This core prediction of the TAP account has received support from imaging studies (Bauch and Otten, 2012; Fellner et al., 2013; Staudigl and Hanslmayr, 2013; Staudigl et al., 2015; Vogelsang et al., 2016, 2018; Long and Kahana, 2017). Here, we replicate and extend the results from these previous studies by showing that the cortical reinstatement of the encoding brain patterns is only associated with beneficial effects for remembering if the replayed patterns are task relevant. Conversely, when the encoding brain patterns were task irrelevant, the cortical reinstatement was associated with detrimental effects on memory performance. Recent work has reported episodic remembering costs, despite a perfect overlap between the encoding and the retrieval contexts, in situations where context does not only overlap with the target episode but also with additional currently irrelevant memory traces (Bramão and Johansson, 2017). The results of the present study are consistent with this previous research by showing that the reinstatement of the encoding brain patterns at retrieval, under certain conditions, can be associated with detrimental effects on memory.

Face-selective cortical processing is known to be reflected in the N170 event-related potential (ERP) component (Rossion and Caharel, 2011), which raises the question whether the early visual category-specific representations here observed are reduce to this face-sensitive mechanism. Our data, notably, speak against this explanation as the predictive value of the early category-specific brain representations was observed for the episodic retrieval of all three picture categories. There was one exception however. In the visual memory task, the early category-specific representations were not predictive of visual retrieval of the landmarks. It is conceivable that discrimination of the target and distracter landmarks might have been influenced by verbal cues to guide selection (e.g., “the tower is to the right”). Such strategies would be harder to implement for faces and objects.

The memory tasks used in this study are hippocampally dependent, and the cortical reinstatement at retrieval of the encoded brain patterns depends on pattern completion operations (Marr, 1971; Norman and O´Reilly, 2003). Previous studies have shown that hippocampal pattern completion and ensuing cortical reinstatement are accomplished ∼500 ms after stimulus onset (Horner et al., 2012; Staresina et al., 2012b; Jafarpour et al., 2014). Others have shown that cortical reinstatement may be more sustained in time for as long as 2000 ms after retrieval cue onset (Johnson et al., 2015). Our data show that pattern completion operations leading to the cortical reinstatement of encoding brain patterns may occur within ∼463 ms after cue onset if the encoding brain patterns carry task-relevant representations. On the other hand, cortical reinstatement of task-irrelevant encoding patterns may occur later during retrieval (∼707 ms after word cue onset). Also, it should be noted that the reinstated activity in the verbal task was formed at later stages of encoding (∼350–420 ms) compared with the visual task (∼120–320 ms). Although the timing of these encoding patterns is different, the consistent topography throughout the epoch suggests that the classification accuracy was based on sustained visual processing. The MVPA may not have captured category-specific representations reflecting lexical processing relevant for the verbal memory task, which may explain the lack of a positive association between classification accuracy and performance in the verbal memory task. Although we cannot offer a conclusive account for the timing differences of replay in the two memory tasks, our results indicate an important role of task requirement and demands in, at least, the timing of the cortical reinstatement.

The topographical distribution of the replay of the encoding brain patterns (Fig. 7*C*) differs from the topography of the category-specific representations observed at encoding (Fig. 3). While this finding aligns well with recent research demonstrating that retrieval may involve the reinstatement of a transformed representation of the encoded information (Xiao et al., 2017), further research is needed to fully understand the functional significance of these transformations. One interesting possibility is that they reflect memory reconstruction mechanisms (i.e., “recontextualization”) leading to a neural representation that varies in the degree of overlap with details of the original episode (Yassa and Reagh, 2013). Similarly, it is conceivable that cortical reinstatement captured with EEG during episodic remembering represents to a lesser degree the low-level sensory activation evoked by the external stimulus input during encoding. In any case, we may conclude that cortical reinstatement during retrieval not only involves literal replay of the processing that occurred in the previous event.

In summary, this is the first study to examine TAP in a paradigm that allowed participants to freely allocate their attention during encoding to whatever attribute they considered relevant. MVPA revealed encoding-related category-specific neural patterns that were replayed at retrieval, and that predicted episodic remembering. Extending the TAP account, we show that the processing engaged during encoding may be associated with both retrieval success and failure depending on the match with later retrieval requirements, thus highlighting also transfer “inappropriate” processing. The present results inform current cognitive neuroscience theories of memory by shedding new light on encoding and retrieval interactions in episodic memory.

## Synthesis

Reviewing Editor: Morgan Barense, University of Toronto Decisions are customarily a result of the Reviewing Editor and the peer reviewers coming together and discussing their recommendations until a consensus is reached. When revisions are invited, a fact-based synthesis statement explaining their decision and outlining what is needed to prepare a revision will be listed below. Note: If this manuscript was transferred from JNeurosci and a decision was made to accept the manuscript without peer review, a brief statement to this effect will instead be what is listed below.

We solicited feedback from the earlier Journal of Neuroscience reviewer. We were both very pleased that this revision carefully considered and addressed all comments.

There were no additional comments, except to congratulate the authors on a nice paper!
